# Dexmedetomidine Attenuates Monocyte-Endothelial Adherence via Inhibiting Connexin43 on Vascular Endothelial Cells

**DOI:** 10.1155/2020/7039854

**Published:** 2020-02-10

**Authors:** Yunfei Chai, Runying Yu, Yong Liu, Sheng Wang, Dongdong Yuan, Jimei Chen

**Affiliations:** ^1^Anesthesiology Department of Guangdong Cardiovascular Institute, Guangdong Provincial People's Hospital, Guangdong Academy of Medical Sciences, Guangzhou, 510080 Guangdong, China; ^2^Operating Centre of Guangdong Provincial People's Hospital, Guangdong Academy of Medical Sciences, Guangzhou, 510080 Guangdong, China; ^3^Department of Cardiology, Guangdong Cardiovascular Institute, Guangdong Provincial Peoples Hospital, Guangdong Academy of Medical Sciences, Guangzhou, 510080 Guangdong, China; ^4^Department of Anesthesiology, Guangdong Provincial People's Hospital, Guangdong Academy of Medical Sciences, Guangzhou, 510080 Guangdong, China; ^5^Department of Anesthesiology, The Third Affiliated Hospital of Sun Yat-Sen University, Tianhe Road, Guangzhou, China; ^6^Department of Cardiovascular Surgery, Guangdong Cardiovascular Institute, Guangdong Provincial People's Hospital, Guangdong Academy of Medical Sciences, Guangzhou, 510080 Guangdong, China

## Abstract

Current studies have identified the multifaceted protective functions of dexmedetomidine on multiple organs. For the first time, we clarify effects of dexmedetomidine on monocyte-endothelial adherence and whether its underlying mechanism is relative to connexin43 (Cx43), a key factor regulating monocyte-endothelial adherence. U937 monocytes and human umbilical vein endothelial cells (HUVECs) were used to explore monocyte-endothelial adherence. Two special siRNAs were designed to knock down Cx43 expression on HUVECs. U937-HUVEC adhesion, adhesion-related molecules, and the activation of the MAPK (p-ERK1/2, p-p38, and p-JNK1/2) signaling pathway were detected. Dexmedetomidine, at its clinically relevant concentrations (0.1 nM and 1 nM), was given as pretreatments to HUVECs. Its effects on Cx43 and U937-HUVEC adhesion were also investigated. The results show that inhibiting Cx43 on HUVECs could attenuate the contents of MCP-1, soluble ICAM-1 (sICAM-1), soluble VCAM-1 (sVCAM-1), and the nonprocessed variants of the adhesion molecules ICAM-1 and VCAM-1 and ultimately result in U937-HUVEC adhesion decrease. Meanwhile, the activation of MAPKs was also inhibited. U0126 (inhibiting p-ERK1/2) and SB202190 (inhibiting p38) decreased the contents of MCP-1, sICAM-1, and sVCAM-1, but SP600125 (inhibiting p-JNK1/2) had none of these effects. ICAM-1 and VCAM-1 could be regulated in a similar way. Dexmedetomidine pretreatment inhibited Cx43 on HUVECs, the activation of MAPKs, and U937-HUVEC adhesion. Therefore, we conclude that dexmedetomidine attenuates U937-HUVEC adhesion via inhibiting Cx43 on HUVECs modulating the activation of MAPK signaling pathways.

## 1. Introduction

Endothelial dysfunction contributes to the development of both acute inflammatory disease states, such as sepsis and endotoxemia, and chronic inflammatory disease states, such as atherosclerosis, rheumatoid arthritis, diabetes, and inflammatory bowel disease [1, 2]. In response to inflammatory stimuli, the vascular endothelium expresses a series of adhesion molecules that play key roles in the recruitment of monocytes to sites of inflammation [3]. These adhesion molecules mediate early monocyte attachment and rolling events. After these events, an inflammatory response within tissues is subsequently generated, such as firm adhesion and transmigration [4]. Some clinical studies have found that the soluble forms of these adhesion molecules, such as sICAM-1 and sVCAM-1, could warn off a series of vascular diseases [5]. Therefore, inhibition-related adhesion molecule expression and monocyte-endothelial adherence might be potent strategies to protect against inflammatory vascular disease.

Connexins are a large family of proteins, expressing in almost all human organs and tissues. They form channels between the neighboring cells, called gap junction, mediating intercellular movement of cytosolic signaling molecules [6]. There are three predominant connexins expressed in endothelial cells, Cx37, Cx40, and Cx43, the most important one of which is Cx43 [7]. Our previous studies demonstrated that Cx43 could regulate monocyte-endothelial adherence via affecting adhesion molecules, firstly indicating the importance of Cx43 in inflammatory vascular disease [8]. In the present study, we focus on effects of Cx43 on the early warning markers of inflammatory vascular disease, such as sICAM-1 and sVCAM-1, in order to find early intervention targets for the treatment of vascular inflammatory diseases. One of the most widely reported signaling pathways relative to monocyte-endothelial adherence is the MAPK signaling pathway, which always results in inflammatory reaction and the production of adhesion molecules [9–11]. Thus, in the present study, we explored whether alternation of Cx43 expression on HUVECs could regulate monocyte-endothelial adherence via mediating the MAPK signaling pathway.

Dexmedetomidine is a highly selective alpha-2 adrenoceptor agonist with sedative, analgesic, and anesthetic effects [12]. Current studies have identified the multifaceted protective functions of dexmedetomidine on multiple organs, such as the heart, nerve, kidney, and liver [13–15]. However, there remains a lack of evidence whether dexmedetomidine could regulate monocyte-endothelial adherence. For the first time, we explored effects of dexmedetomidine on monocyte-endothelial adherence.

In the clinical setting, dexmedetomidine is widely employed in anesthesia and intensive care units [16]. Patients undergoing operation or staying in the intensive care units experience a long period of supine position, which lead to hemodynamic changes. Under these circumstances, monocytes flowing in blood vessels will be easy to adhere to the inflamed or damaged vascular endothelial cells, aggravating vascular injury or thrombosis [17, 18]. This is detrimental to the patient's recovery. Therefore, through the study of dexmedetomidine, we hope to find a new method to protect against monocyte-endothelial adherence and more importantly elucidate a novel mechanism underlying the effects of analgesics in counteracting inflammatory vascular disease.

## 2. Material and Methods

### 2.1. Cell Culture

The present study protocol conformed to the ethical guidelines of the 1975 Declaration of Helsinki with the approval of the Institutional Medical Ethics Committee of the Third Affiliated Hospital of Sun Yat-sen University. Both U937 monocytes and HUVECs are obtained from American Type Culture Collection (Manassas, VA, USA). HUVECs are cultured with human endothelial SFM (Invitrogen, Carlsbad, CA, USA), containing 20% fetal bovine serum (Invitrogen), 100 U/ml penicillin-streptomycin (Invitrogen), 100 *μ*g/ml heparin (Sigma-Aldrich, St. Louis, MO, USA), and 150 *μ*g/ml endothelial cell growth supplement (Becton, Dickinson and Company, Franklin Lakes, NJ, USA). U937 monocytes are cultured in RPMI1640 medium (Invitrogen), containing 20% fetal bovine serum (Invitrogen) and 100 U/ml penicillin-streptomycin (Invitrogen). These two cell lines are both cultured in a 5% CO_2_ incubator (37°C and 90% humidity) (Thermo Fisher Scientific, Waltham, MA, USA).

### 2.2. Inhibition of Cx43 Expression by Small Interfering RNA (siRNA) Transfection [6]

We design two special siRNAs (Cx43-siRNA1: GCTGGTTACTGGTGACAGA; Cx43-siRNA2: CCGCAATTACAACAAGCAA) targeting Cx43 gene and a nonspecific, control siRNA (NC; CGCGATATAGCGCATCGAT) to inhibit Cx43 expression on HUVECs. Transfection into HUVECs is carried out using Lipofectamine 2000 (Invitrogen, Carlsbad, CA, USA), according to the manufacturer's instructions.

### 2.3. Parachute Dye-Coupling Assay [19]

A parachute dye-coupling assay is always used to examine function of gap junction intercellular communication. In the present study, the parachute dye-coupling assay is used to examine gap junction intercellular communication composed of Cx43 on HUVECs. Briefly, HUVECs grow to confluence. Donor cells are labeled with 5 *μ*mol/l calcein-AM (30 minutes at 37°C). After that, the donor cells are trypsinized and seeded onto the receiver cells at a ratio of 1 : 150 (donor : receiver). The donor cells are allowed to attach to the monolayer of the receiver cells to form gap junctions for 4 hours at 37°C. The results are observed and counted with a fluorescence microscope (Olympus DP73, Tokyo, Japan). For each well, 8 different 200x visual fields in the middle of the dish are chosen for analysis. The average number of receiver cells around every donor cell is recorded by an experienced researcher who do not know the groups. We consider the data of the control group as 1, and other groups are normalized to the control group.

### 2.4. Cell Treatments

Dexmedetomidine is purchased from Hengrui Medicine (Jiangsu, China). Before other tests, HUVECs are pretreated with Dexm (0.1 nM and 1 nM) for 24 hours. U0126 was used to inhibit p-ERK1/2 (10 *μ*M, 24 hours, Sigma-Aldrich); SB202190 was used to inhibit p-p38 (10 *μ*M, 24 hours, Sigma-Aldrich); SP600125 was used to inhibit p-JNK1/2 (10 *μ*M, 24 hours, Sigma-Aldrich).

### 2.5. Adhesion Assay [2]

U937 monocytes are labeled with 5 *μ*mol/l calcein-acetoxymethyl ester (Invitrogen) and cultured in the incubator for 30 min. Then, the labeled cells are washed twice with PBS (Invitrogen) and resuspended in the medium without serum. After that, the labeled cells are added onto confluent monolayers of HUVECs, pretreated with recombinant mouse tumor necrosis factor (TNF-*α*, 10 ng/ml, Peprotech, Rocky Hill, NJ, USA) for 12 hours. The plates are put back into the incubator. After 1-hour incubation, the plates were rinsed twice slightly with medium without serum. Adherent U937 monocytes are left and remain on the confluent monolayers of HUVECs. The adherent U937 monocytes are counted with a fluorescence microscope (Olympus IX71, Tokyo, Japan). For each well, 8 different 200x visual fields in the middle of the dish are chosen for analysis. The average number of receiver cells around every donor cell is recorded by an experienced researcher who do not know the groups. We consider the data of the control group as 1, and other groups are normalized to the control group. The normalized data is just adhesion fraction.

### 2.6. Protein Detection

MCP-1, sICAM-1, and sVCAM-1 in supernatants are detected with an ELISA kit (Sigma-Aldrich) according to the instructions.

Cx43, VCAM-1, ICAM-1, p-ERK1/2, p-p38, and p-JNK1/2 are detected with western blotting. The cells are washed with cold PBS and harvested in lysis buffer (Bio-Rad, Hercules, CA), sonicated, and centrifuged at 14167 g for 30 min at 4°C. Protein samples are quantified with a Pierce™ BCA Protein Assay Kit (Thermo Fisher Scientific, Inc., Waltham, MA, USA). Subsequently, 25 *μ*g of each protein sample is added into SDS-PAGE and then transferred onto a polyvinylidene fluoride membrane. Membranes are blocked with 5% milk for 1 hour at room temperature and incubated with the primary antibodies overnight at 4°C. The dilution of antibodies is as following: anti-Cx43 (1 : 3000, Sigma-Aldrich), VCAM-1 (Santa Cruz Biotechnology, Santa Cruz, CA, USA), ICAM-1 (Santa Cruz Biotechnology), p-ERK and ERK (1 : 3000, Cell Signaling Technology, Inc., Danvers, MA, USA), p-p38 and p38 (1 : 3000, Cell Signaling Technology, Inc.), p-JNK and JNK (1 : 3000, Cell Signaling Technology, Inc.), and anti-*β*-tubulin (1 : 10000, Sigma-Aldrich). Protein band sizes are estimated using Alpha View software (version number: 2.2.14407, ProteinSimple, Santa Clara, CA, USA).

### 2.7. Statistical Analysis

Statistical analysis is performed by using SPSS 15.0 software (SPSS, Inc., Chicago, IL, USA). Multiple comparisons among groups are analyzed using one-way ANOVA, followed by Tukey post hoc comparisons. The data are presented as the mean ± SEM.

## 3. Results

After we have shown that all effects investigated in this work were induced by TNF-*α* (Supplemental [Supplementary-material supplementary-material-1]), we only worked with TNF-*α*-pretreated cells in the following results.

### 3.1. Inhibiting Cx43 on HUVECs Could Attenuate U937-HUVEC Adhesion

**Figure 1:**
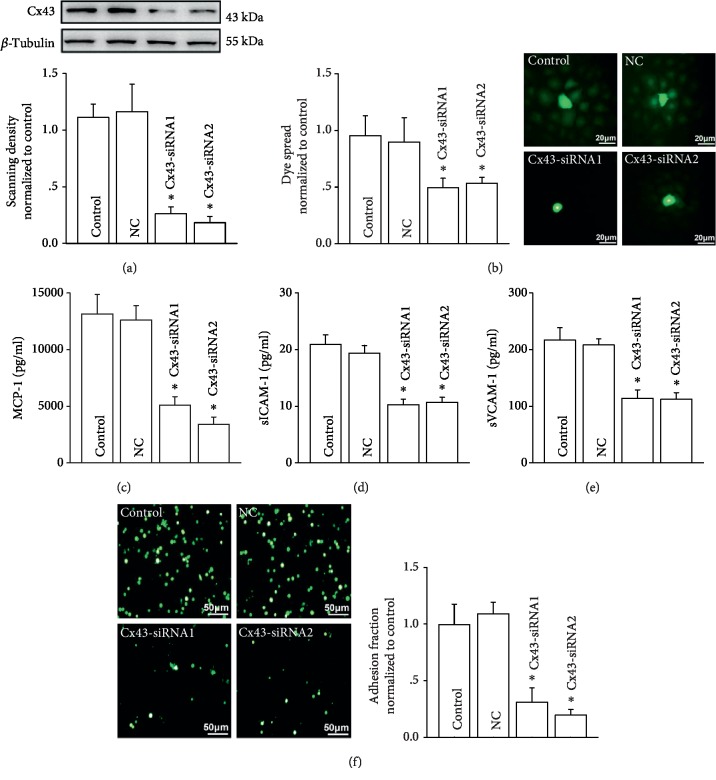
Inhibiting Cx43 could attenuate U937-HUVEC adhesion, as well as MCP-1, sICAM-1, and sVCAM-1. (a) Expression of Cx43 on HUVECs following treatment with two kinds of Cx43-siRNAs (*n* = 4, ^∗^*P* < 0.05*vs.* control). (b) Effects of the two kinds of Cx43-siRNAs on dye coupling (*n* = 6, ^∗^*P* < 0.05*vs.* control). (c–e) The contents of MCP-1, sICAM-1, and sVCAM-1 when HUVECs were pretreated with Cx43-siRNAs (*n* = 5, ^∗^*P* < 0.05*vs.* control). (f) The changes of U937-HUVEC adhesion when HUVECs were pretreated with Cx43-siRNAs (*n* = 5, ^∗^*P* < 0.05*vs.* control). Control group means lipofectamine 2000 pretreatment group; NC: negative control (negative control has random base sequence that is different from Cx43-siRNA1 and Cx43-siRNA1). In all experiments, HUVECs were all pretreated with TNF-*α* (10 ng/ml, 12 h). Effects of TNF-*α* are showed in Supplemental [Supplementary-material supplementary-material-1].

To investigate effects of Cx43 on U937-HUVEC adhesion, we designed two special siRNAs targeting Cx43 gene (Cx43-siRNA1 and Cx43-siRNA2) to knock down Cx43 expression on HUVECs.  Figure 1(a) showed that both of the two siRNAs could attenuate Cx43 expression on HUVECs significantly. Meanwhile, dye spread between the neighboring cells was also reduced, which reflects functions of the gap junction composed of Cx43 (Figure 1(b)).

We then evaluated the contents of MCP-1, sICAM-1, and sVCAM-1, all of which functioned in U937-HUVEC adhesion. As shown in Figures 1(c)–1(e), the contents of these molecules were all decreased as Cx43 expression was knocked down by siRNAs. According to the reports, we notice that sICAM-1 and sVCAM-1 are always considered to be the inflammatory markers and their nonprocessed variants, ICAM-1 and VCAM-1, are responsible for monocyte adhesion. Therefore, we also detected effects of Cx43 inhibition on ICAM-1 and VCAM-1 expression, and the results showed that ICAM-1 and VCAM-1 expression was attenuated significantly (Supplemental [Supplementary-material supplementary-material-1]). More importantly, U937-HUVEC adhesion was downregulated obviously (Figure 1(f)).

### 3.2. Inhibiting Cx43 on HUVECs Could Attenuate U937-HUVEC Adhesion via Modulating MAPK Signaling Pathway

**Figure 2:**
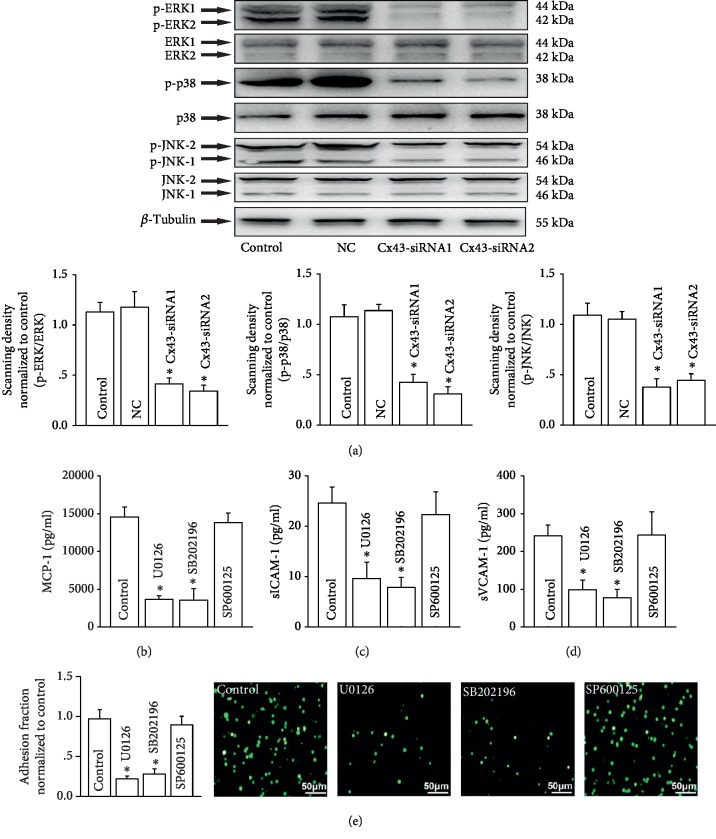
Inhibiting Cx43 on HUVECs could attenuate U937-HUVEC adhesion via modulating the MAPK signaling pathway. (a) Effects of two kinds of Cx43-siRNAs on the activation of MAPKs (p-ERK/ERK, p-p38/p38, and p-JNK/JNK) (*n* = 4, ^∗^*P* < 0.05*vs.* control). Control group means lipofectamine 2000 pretreatment group; NC: negative control (negative control has random base sequence that differs from Cx43-siRNA1 and Cx43-siRNA1). (b–d) The contents of MCP-1, sICAM-1, and sVCAM-1 when HUVECs were pretreated with U0126 (inhibiting p-ERK1/2, 10 *μ*M, 24 hours), SB202190 (inhibiting p38, 10 *μ*M, 24 hours), and SP600125 (inhibiting p-JNK1/2, 10 *μ*M, 24 hours) (*n* = 5, ^∗^*P* < 0.05*vs.* control). (e) The changes of U937-HUVEC adhesion when HUVECs were pretreated with U0126, SB202190, and SP600125 (*n* = 5, ^∗^*P* < 0.05*vs.* control). The solvents of U0126, SB202190, and SP600125 were DMSO, which had no effects on the above results. (b–e) The control group is just DMSO pretreatment. In all experiments, HUVECs were all pretreated with TNF-*α* (10 ng/ml, 12 h). Effects of TNF-*α* are showed in Supplemental [Supplementary-material supplementary-material-1].

One of the most widely reported signaling pathway relative to monocyte-endothelial adherence is the MAPK signaling pathway [20]. Therefore, we detected effects of Cx43 on this signaling pathway.  Figure 2(a) showed that inhibiting Cx43 expression on HUVECs with Cx43-siRNAs could attenuate the activation of MAPKs, manifested as the decrease of p-ERK1/2, p-p38, and p-JNK expression. Furthermore, MAPK inhibitors, U0126 (inhibiting p-ERK1/2) and SB202190 (inhibiting p38), decreased the contents of MCP-1, sICAM-1, sVCAM-1, ICAM-1, and VCAM-1, but SP600125 (inhibiting p-JNK1/2) had none of these effects (Figures 2(b)–2(d); Supplemental Figures [Supplementary-material supplementary-material-1]C and [Supplementary-material supplementary-material-1]D), which might be the reason why inhibiting p-JNK1/2 with SP600125 had no effect on U937-HUVEC adhesion (Figure 2(e)). Results above indicated that inhibiting Cx43 expression on HUVECs could attenuate U937-HUVEC adhesion via decreasing the activation of the MAPK signaling pathway.

### 3.3. Dexmedetomidine Could Inhibit Gap Junction Function and Cx43 Expression on HUVECs

**Figure 3:**
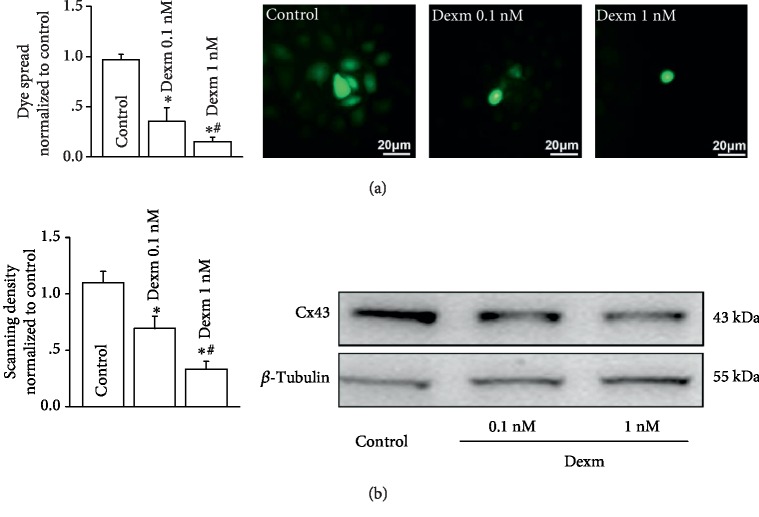
Dexmedetomidine inhibited gap junction function and Cx43 expression on HUVECs. (a) Effects of dexmedetomidine (Dexm: 0.1 nM and 1 nM, 24 hours) on dye coupling on HUVECs (*n* = 4, ^∗^*P* < 0.05*vs.* control; ^#^*P* < 0.05*vs.* 0.1 nM group). (b) Effects of dexmedetomidine (Dexm: 0.1 nM and 1 nM, 24 hours) on Cx43 expression on HUVECs (*n* = 4, ^∗^*P* < 0.05*vs.* control; ^#^*P* < 0.05*vs.* 0.1 nM group). In all experiments, HUVECs were all pretreated with TNF-*α* (10 ng/ml, 12 h). Effects of TNF-*α* are showed in Supplemental [Supplementary-material supplementary-material-1].

When HUVECs were exposed to clinically relevant concentrations (0.1 nM and 1 nM) of dexmedetomidine for 24 hours, the function of the gap junction and Cx43 expression were both reduced. Inhibitory effects of 1 nM dexmedetomidine on gap junction function or Cx43 expression were more significant than 0.1 nM dexmedetomidine (Figure 3).

### 3.4. Dexmedetomidine Attenuated U937-HUVEC Adhesion, as well as the Contents of MCP-1, sICAM-1, and sVCAM-1

**Figure 4:**
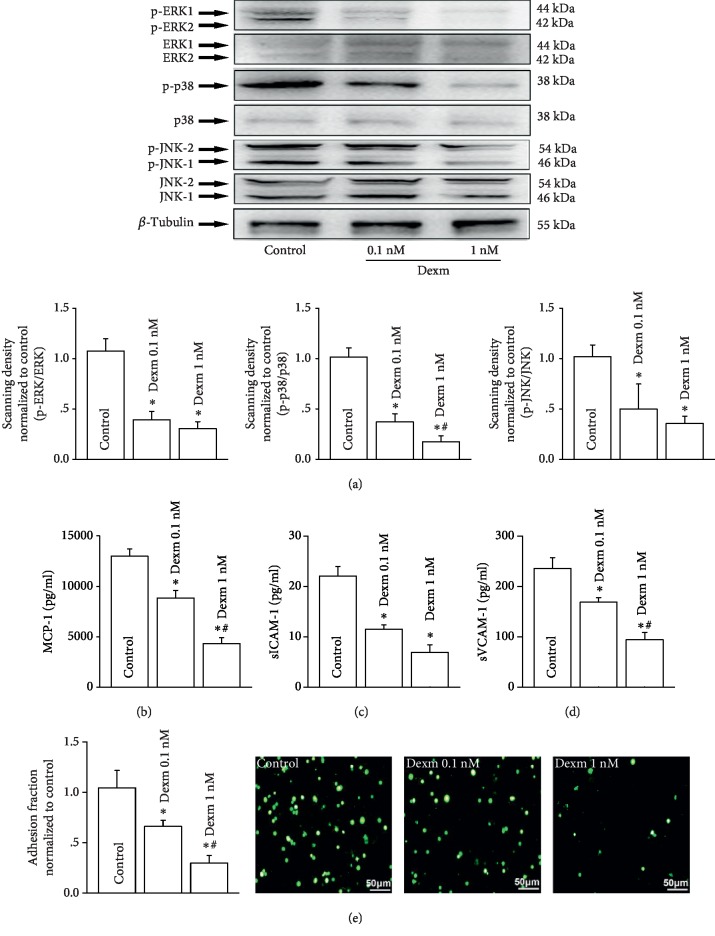
Dexmedetomidine attenuated U937-HUVEC adhesion, as well as the contents of MCP-1, sICAM-1, and sVCAM-1. (a) Effects of dexmedetomidine (Dexm: 0.1 nM and 1 nM, 24 hours) on the activation of MAPKs (p-ERK/ERK, p-p38/p38, and p-JNK/JNK) (*n* = 4, ^∗^*P* < 0.05*vs.* control). (b–d) The contents of MCP-1, sICAM-1, and sVCAM-1 when HUVECs were pretreated with dexmedetomidine (Dexm: 0.1 nM and 1 nM, 24 hours) (*n* = 4, ^∗^*P* < 0.05*vs.* control; ^#^*P* < 0.05*vs.* 0.1 nM group). (e) The changes of U937-HUVEC adhesion when HUVECs were pretreated with dexmedetomidine (Dexm: 0.1 nM and 1 nM, 24 hours) (*n* = 4, ^∗^*P* < 0.05*vs.* control; ^#^*P* < 0.05*vs.* 0.1 nM group). In all experiments, HUVECs were all pretreated with TNF-*α* (10 ng/ml, 12 h). Effects of TNF-*α* are showed in Supplemental [Supplementary-material supplementary-material-1].

Combined with the results that inhibiting Cx43 on HUVECs could attenuate U937-HUVEC adhesion via decreasing the activation of the MAPK signaling pathway (Figures 1 and 2) and dexmedetomidine pretreatment could inhibit Cx43 on HUVEC (Figure 3), we speculated that dexmedetomidine could also reduce U937-HUVEC adhesion via inhibiting Cx43, the mechanism of which might be relative to MAPK signaling pathway regulation.  Figure 4(a) demonstrated that dexmedetomidine pretreatment (0.1 nM and 1 nM) on HUVECs for 24 hours could also attenuate the activation of the MAPK signaling pathway: all of p-ERK1/2, p-p38, and p-JNK1/2 expressions were downregulated. Meanwhile, the contents of relative adhesion molecules, such as MCP-1, sICAM-1, sVCAM-1, ICAM-1, and VCAM-1, were also reduced obviously because of dexmedetomidine pretreatment (Figures 4(b)–4(d); Supplemental [Supplementary-material supplementary-material-1]). Ultimately, U937-HUVEC adhesion was also attenuated (Figure 4(e)).

## 4. Discussion

Monocyte-endothelial adherence is always closely related to the occurrence and development of many vascular pathologies, including acute coronary syndromes, diabetic nephropathy, bacterial endocarditis, and atherosclerosis [1]. Adherent monocytes release a large number of chemoattractants and inflammatory factors, which not only self-reinforce cell adhesion but also furtherly damage the vascular endothelium [7]. Therefore, understanding the underlying mechanism of monocyte-endothelial adherence could help us to take effective strategies for the prevention and treatment of inflammatory vascular diseases.

The present study demonstrated that inhibiting Cx43 on HUVECs could attenuate the contents of adhesion-related molecules, such as MCP-1, sICAM-1, sVCAM-1, ICAM-1, and VCAM-1, and ultimately result in U937-HUVEC adhesion decrease. Dexmedetomidine pretreatment for 24 hours at its clinically relevant concentrations (0.1 nM and 1 nM) also could reduce U937-HUVEC adhesion via inhibiting Cx43 on HUVECs. This revealed a novel mechanism underlying the effects of analgesics in counteracting monocyte-endothelial adherence.

There are three predominant connexins expressed in endothelial cells, Cx37, Cx40, and Cx43, among which Cx43 is the most widespread connexin protein in the cardiovascular system and plays an important part in normal physiology and cardiovascular pathologies [21]. Our results show that inhibiting Cx43 could attenuate U937-HUVEC adhesion and related adhesion molecules, such as MCP-1, sICAM-1, sVCAM-1, ICAM-1, and VCAM-1. MCP-1 is the first discovered and most extensively studied CC chemokine. It has a strong chemotactic effect on monocytes, inducing the migration, aggregation, adhesion, and activation of monocytes to the injury site of the arterial wall and ultimately resulting in vascular injury [22]. sICAM-1 and sVCAM-1 are circulating soluble forms of homonymous endothelial cell surface molecules modulating the adhesion and migration of monocytes with the help of integrins VLA-4 and LFA-1 [23]. As reported, sICAM-1 and sVCAM-1 could warn off a series of vascular diseases, including atherosclerosis [24, 25]. We firstly demonstrated that both of the two early warning markers could be regulated by Cx43. As far as we know, sICAM-1 and sVCAM-1 are just the processed variants of the adhesion molecules ICAM-1 and VCAM-1. sICAM-1 and sVCAM-1 are always considered to be the inflammatory markers, and the nonprocessed variants, ICAM-1 and VCAM-1, are responsible for monocyte adhesion. Our supplemental results showed that the changes of ICAM-1 and VCAM-1 are the same with that of sICAM-1 and sVCAM-1, all of which could be regulated by Cx43 (Supplemental [Supplementary-material supplementary-material-1]). These results prompt us that Cx43 might be a potential target preventing against vascular injury via downregulating MCP-1, sICAM-1, sVCAM-1, ICAM-1, and VCAM-1 expression, even better than MCP-1, sICAM-1, sVCAM-1, ICAM-1, and VCAM-1 themselves.

One of the most widely reported signaling pathways relative to monocyte-endothelial adherence is the MAPK signaling pathway, which always results in inflammatory reaction and the production of adhesion molecules [26–28]. The important finding in the present study is that alternation of Cx43 expression on HUVECs modulated the activation of MAPKs, manifested as the changes of p-ERK1/2, p-p38, and p-JNK1/2. The carboxyl-terminal domain of Cx43 could interact with some special elements of cellular signaling pathways, such as Src, PKC, and PKA, which provides the possibility that changes of Cx43 expression affect other signaling pathways [2, 8, 29]. Although inhibiting Cx43 expression on HUVECs could effectively attenuate the activation of ERK1/2, p38, and JNK1/2, it did not mean that all the three signaling pathways could affect related adhesion molecule expression and cell adhesion. Our results in Figures 2(b)–2(d) and Supplemental Figures [Supplementary-material supplementary-material-1]C and [Supplementary-material supplementary-material-1]D just showed that U0126 (inhibiting p-ERK1/2) and SB202190 (inhibiting p38) decreased the contents of MCP-1, sICAM-1, sVCAM-1, ICAM-1, and VCAM-1, but SP600125 (inhibiting p-JNK1/2) had none of these effects. That might be the reason why inhibiting p-JNK1/2 with SP600125 had no effect on U937-HUVEC adhesion (Figure 2(e)). These findings indicated that the JNK1/2 signaling pathway had nothing to do with expression of MCP-1, sICAM-1, sVCAM-1, ICAM-1, and VCAM-1. Some of these observations are identical to the findings reported by Bian et al. and Cheng et al., who showed that only ERK1/2 or p38 inhibitors were able to reduce sICAM-1 and MCP-1 levels [30, 31]. We believe that this conclusion provides more precise targets for the intervention of monocyte-endothelial adherence.

Under normal conditions, circulating monocytes interact minimally with vascular endothelial cells [32]. Patients undergoing operation or staying in the intensive care units will experience a long term of supine position. Hemodynamics will be significantly affected. Monocytes flowing in blood vessels will easily adhere to the inflamed or damaged vascular endothelial cells, aggravating vascular injury or thrombosis [33]. This is detrimental to the patient's recovery. Therefore, it is very imperative to find potent strategies to avoid monocyte-endothelial adherence. Dexmedetomidine, a highly selective alpha-2 adrenoceptor agonist with sedative, analgesic, and anesthetic effects, has been widely used in anesthesia and intensive care. Its protective effects on multiple organs, such as the heart, nerve, kidney, and liver, have been widely reported by different researchers [12]. Nevertheless, the relationship between dexmedetomidine and monocyte-endothelial adherence has never been explored. For the first time, we clarified that dexmedetomidine at its clinically relevant concentrations (0.1 nM and 1 nM) could attenuate monocyte-endothelial adherence, as well as some related adhesion molecules, the mechanism of which was relative to its inhibitory effect on Cx43 on HUVECs. Certainly, we understand that it is not that simple to extrapolate the findings of an *in vitro* study to the clinical setting, but, at least, the present results provided us a possible method to resolve this problem under the condition that dexmedetomidine has been widely used in clinic and accepted by physicians and anesthesiologists. The lack of relevant clinical research is just the limitation of this subject.

## 5. Conclusion

The present study demonstrated that Cx43 might be a potent target against monocyte-endothelial adherence, even better than some early warning markers, such as MCP-1, sICAM-1, sVCAM-1, ICAM-1, and VCAM-1, because it could regulate this early warning marker expression. Its possible mechanism was relative to Cx43 alternation regulating the activation of MAPK signaling pathways. Dexmedetomidine pretreatment at its clinically relevant concentrations (0.1 nM and 1 nM) could reduce monocyte-endothelial adherence via inhibiting Cx43 on HUVECs, providing an effective strategy against monocyte-endothelial adherence in clinic.

## Data Availability

The data used to support the findings of this study are included within the article.

## Supplementary Materials

Supplementary Materials TNF-*α* pretreatment could increase Cx43 expression on HUVECs, U937-HUVEC adhesion, adhesion-related molecules, and active MAPK signaling pathway. In Supplemental Figure 1, we explored effects of TNF-*α* on Cx43, adhesion-related molecules, U937-HUVEC adhesion, and MAPK signaling pathway. Supplemental Figures 1A and 1B showed that when HUVECs were pretreated with TNF-*α*, both Cx43 expression and its function were increased obviously. Meanwhile, adhesion-related molecules (such as MCP-1, sICAM-1, and sVCAM-1) and U937-HUVEC adhesion were both increased (Supplemental Figures 1C to 1F). Finally, we tested the changes of the MAPK signaling pathway and found that the MAPK signaling pathway was activated by TNF-*α* pretreatment, manifested as the expression of p-ERK, p-p38, and p-JNK which were increased, but the total ERK, p38, and JNK had no changes (Supplemental Figure 1G). Both Cx43 expression inhibition and dexmedetomidine pretreatment could attenuate VCAM-1 and ICAM-1 expression induced by TNF-*α* on HUVECs. Supplemental Figures 2A and 2B showed that TNF-*α* pretreatment could induce VCAM-1 and ICAM-1 expression on HUVECs effectively, which could be inhibited by Cx43-siRNA and dexmedetomidine. Combined with the results that dexmedetomidine could attenuate Cx43 expression on HUVECs in Figure 3, we conclude that dexmedetomidine could inhibit VCAM-1 and ICAM-1 expression via attenuating Cx43 expression on HUVECs. U0126 and SB202190 but not SP600125 could also attenuate VCAM-1 and ICAM-1 expression induced by TNF-*α* on HUVECs. In Supplemental Figures 2C and 2D, we found that U0126 and SB202190 but not SP600125 could also attenuate VCAM-1 and ICAM-1 expression induced by TNF-*α* on HUVECs. These results were consistent with the changes of adhesion-related molecules (such as MCP-1, sICAM-1, and sVCAM-1) and U937-HUVEC adhesion in Figures 2(b)–2(e). These results showed that ERK and p38 but not JNK could affect adhesion-related molecules (such as MCP-1, sICAM-1, ICAM-1, sVCAM-1, and VCAM-1) and U937-HUVEC adhesion, which provided us more precise targets for the intervention of monocyte-endothelial adherence. Supplemental Figure 1: effects of TNF-*α* on Cx43, adhesion-related molecules, U937-HUVEC adhesion, and MAPK signaling pathway. (A) Effects of TNF-*α* (10 ng/ml, 12 h) on Cx43 expression (*n* = 3, ^∗^*P* < 0.05 vs. without TNF-*α* group); (B) effects of TNF-*α* (10 ng/ml, 12 h) on dye coupling (*n* = 3, ^∗^*P* < 0.05 vs. without TNF-*α* group); (C-E) the contents of MCP-1, sICAM-1, and sVCAM-1 when HUVECs were pretreated with TNF-*α* (10 ng/ml, 12 h) (*n* = 3, ^∗^*P* < 0.05 vs. without TNF-*α* group); (F) the changes of U937-HUVEC adhesion when HUVECs were pretreated with TNF-*α* (10 ng/ml, 12 h) (*n* = 3, ^∗^*P* < 0.05 vs. without TNF-*α* group); (G) effects of TNF-*α* (10 ng/ml, 12 h) on the activation of MAPKs (p-ERK/ERK, p-p38/p38, and p-JNK/JNK) (*n* = 3, ^∗^*P* < 0.05 vs. without TNF-*α* group). Supplemental Figure 2: effects of Cx43-siRNA, dexmedetomidine, and MAPK signaling pathway on VCAM-1 and ICAM-1 expression on HUVECs. (A, B) Effects of Cx43-siRNA and dexmedetomidine (Dexm: 0.1 nM and 1 nM, 24 hours) on VCAM-1 and ICAM-1 expression on HUVECs (*n* = 3, ^∗^*P* < 0.05 vs. control); (C, D) the changes of VCAM-1 and ICAM-1 expression when HUVECs were pretreated with U0126 (inhibiting p-ERK1/2, 10 *μ*M, 24 hours), SB202190 (inhibiting p38, 10 *μ*M, 24 hours), and SP600125 (inhibiting p-JNK1/2, 10 *μ*M, 24 hours) (*n* = 3, ^∗^*P* < 0.05 vs. control).
