# Supplementary material for: Dexmedetomidine Attenuates Monocyte-Endothelial Adherence via Inhibiting Connexin43 on Vascular Endothelial Cells
Source: Mediators Inflamm. 2020 Feb 10;2020:7039854. doi: 10.1155/2020/7039854 (PMC7035564; doi:10.1155/2020/7039854)
Supplement: Supplementary Materials — TNF-α pretreatment could increase Cx43 expression on HUVECs, U937-HUVEC adhesion, adhesion-related molecules, and active MAPK signaling pathway. In Supplemental Figure 1, we explored effects of TNF-α on Cx43, adhesion-related molecules, U937-HUVEC adhesion, and MAPK signaling pathway. Supplemental Figures 1A and 1B showed that when HUVECs were pretreated with TNF-α, both Cx43 expression and its function were increased obviously. Meanwhile, adhesion-related molecules (such as MCP-1, sICAM-1, and sVCAM-1) and U937-HUVEC adhesion were both increased (Supplemental Figures 1C to 1F). Finally, we tested the changes of the MAPK signaling pathway and found that the MAPK signaling pathway was activated by TNF-α pretreatment, manifested as the expression of p-ERK, p-p38, and p-JNK which were increased, but the total ERK, p38, and JNK had no changes (Supplemental Figure 1G). Both Cx43 expression inhibition and dexmedetomidine pretreatment could attenuate VCAM-1 and ICAM-1 expression induced by TNF-α on HUVECs. Supplemental Figures 2A and 2B showed that TNF-α pretreatment could induce VCAM-1 and ICAM-1 expression on HUVECs effectively, which could be inhibited by Cx43-siRNA and dexmedetomidine. Combined with the results that dexmedetomidine could attenuate Cx43 expression on HUVECs in Figure 3, we conclude that dexmedetomidine could inhibit VCAM-1 and ICAM-1 expression via attenuating Cx43 expression on HUVECs. U0126 and SB202190 but not SP600125 could also attenuate VCAM-1 and ICAM-1 expression induced by TNF-α on HUVECs. In Supplemental Figures 2C and 2D, we found that U0126 and SB202190 but not SP600125 could also attenuate VCAM-1 and ICAM-1 expression induced by TNF-α on HUVECs. These results were consistent with the changes of adhesion-related molecules (such as MCP-1, sICAM-1, and sVCAM-1) and U937-HUVEC adhesion in Figures 2(b)–2(e). These results showed that ERK and p38 but not JNK could affect adhesion-related molecules (such as MCP-1, sICAM-1, ICAM-1, sVCAM-1, and VCA [file 7039854.f1.docx]

**Supplemental materials**

**TNF-α pretreatment could increase Cx43 expression on HUVECs, U937-HUVECs adhesion, adhesion related molecular, and active MAPK signaling pathway.**

In supplemental figure 1, we explored effects of TNF-α on Cx43, adhesion related molecular, U937-HUVECs adhesion and MAPK signaling pathway. Supplemental figure 1A and B showed that when HUVECs were pretreated with TNF-α, both Cx43 expression and its function were increased obviously. Meanwhile, adhesion related molecular (such as MCP-1, sICAM-1 and sVCAM-1) and U937-HUVECs adhesion were both increased (supplemental figure 1C to F). Finally, we tested the changes of the MAPK signaling pathway and found that the MAPK signaling pathway was activated by TNF-α pretreatment, manifested as the expression of p-ERK, p-p38 and p-JNK were increased, but the total ERK, p38 and JNK had no changes (supplemental figure 1G).

**Both Cx43 expression inhibition and dexmedetomidine pretreatment could attenuate VCAM-1 and ICAM-1 expression induced by TNF-α on HUVECs.**

Supplemental figure 2A and B showed that TNF-α pretreatment could induce VCAM-1 and ICAM-1 expression on HUVECs effectively, which could be inhibited by Cx43-siRNA and dexmedetomidine. Combined with the results that dexmedetomidine could attenuate Cx43 expression on HUVECs in figure 3, we conclude that dexmedetomidine could inhibit VCAM-1 and ICAM-1 expression via attenuating Cx43 expression on HUVECs.

**U0126, SB202190 but SP600125 could also attenuate VCAM-1 and ICAM-1 expression induced by TNF-α on HUVECs.**

In supplemental figure 2C and D, we found that U0126, SB202190 but not SP600125 could also attenuate VCAM-1 and ICAM-1 expression induced by TNF-α on HUVECs. These results were consistent with the changes of adhesion related molecular (such as MCP-1, sICAM-1 and sVCAM-1) and U937-HUVECs adhesion in figure 2B to E. These results showed that ERK, p38 but not JNK could affect adhesion related molecular (such as MCP-1, sICAM-1, ICAM-1, sVCAM-1 and VCAM-1) and U937-HUVECs adhesion, which provided us more precise targets for the intervention of monocyte-endothelial adherence.


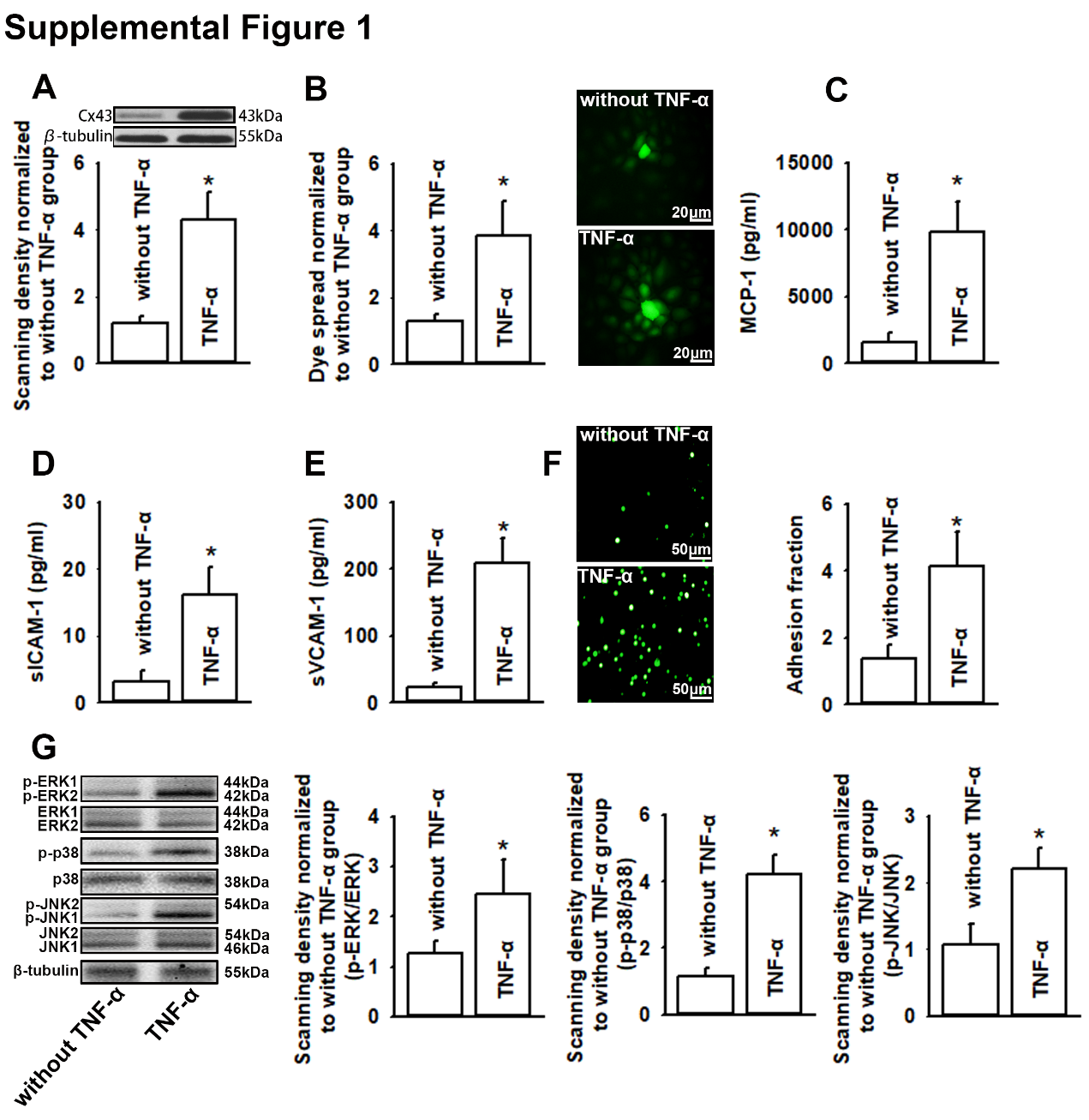


**Supplemental Figure 1. Effects of TNF-α on Cx43, adhesion related molecular, U937-HUVECs adhesion and MAPK signaling pathway.**

(A) Effects of TNF-α (10ng/ml, 12h) on Cx43 expression (n=3, **P* < 0.05 vs without TNF-α group); (B) Effects of TNF-α (10ng/ml, 12h) on dye coupling (n=3, **P* < 0.05 vs without TNF-α group); (C-E) The contents of MCP-1, sICAM-1 and sVCAM-1 when HUVECs were pretreated with TNF-α (10ng/ml, 12h) (n=3, **P* < 0.05 vs without TNF-α group); (F) The changes of U937-HUVECs adhesion when HUVECs were pretreated with TNF-α (10ng/ml, 12h) (n=3, **P* < 0.05 vs without TNF-α group); (G) Effects of TNF-α (10ng/ml, 12h) on the activation of MAPKs (p-ERK/ERK, p-p38/p38 and p-JNK/JNK) (n=3, **P* < 0.05 vs without TNF-α group).





**Supplemental Figure 2. Effects of Cx43-siRNA, dexmedetomidine and MAPK signaling pathway on VCAM-1 and ICAM-1 expression on HUVECs.**

(A-B) Effects of Cx43-siRNA and dexmedetomidine (Dexm: 0.1 nM and 1 nM, 24 hours) on VCAM-1 and ICAM-1 expression on HUVECs (n=3, **P* < 0.05 vs control); (C-D) The changes of VCAM-1 and ICAM-1 expression when HUVECs were pretreated with U0126 (inhibiting p-ERK1/2, 10 μM, 24 hours), SB202190 (inhibiting p38, 10 μM, 24 hours) and SP600125 (inhibiting p-JNK1/2, 10 μM, 24 hours) (n=3, **P* < 0.05 vs control).
